# The maternal environment interacts with genetic variation in regulating seed dormancy in Swedish *Arabidopsis thaliana*

**DOI:** 10.1371/journal.pone.0190242

**Published:** 2017-12-27

**Authors:** Envel Kerdaffrec, Magnus Nordborg

**Affiliations:** Gregor Mendel Institute, Austrian Academy of Sciences, Vienna Biocenter (VBC), Vienna, Austria; Wuhan Botanical Garden, CHINA

## Abstract

Seed dormancy is a complex adaptive trait that controls the timing of seed germination, one of the major fitness components in many plant species. Despite being highly heritable, seed dormancy is extremely plastic and influenced by a wide range of environmental cues. Here, using a set of 92 *Arabidopsis thaliana* lines from Sweden, we investigate the effect of seed maturation temperature on dormancy variation at the population level. The response to temperature differs dramatically between lines, demonstrating that genotype and the maternal environment interact in controlling the trait. By performing a genome-wide association study (GWAS), we identified several candidate genes that could presumably account for this plasticity, two of which are involved in the photoinduction of germination. Altogether, our results provide insight into both the molecular mechanisms and the evolution of dormancy plasticity, and can serve to improve our understanding of environmentally dependent life-history transitions.

## Introduction

Life-stage transitions, the timing of which is critical to plant fitness, are regulated by both genes and the environment, usually in interaction (G x E) [1–3]. Plants have evolved ways to sense and integrate environmental inputs in order to adjust their life cycle to seasonal environments, the best-known example of which is vernalization and the perception of winter cold [4]. In *A. thaliana*, vernalization results in the stable repression of the central regulator *FLOWERING LOCUS C* (*FLC*), a prerequisite for the vegetative-to-reproductive transition to occur [5].

While flowering time determines the reproductive environment, seed dormancy, another major life-history trait crucial for local adaptation, regulates the timing of germination and determines the post-germination environment [1, 6, 7]. Dormancy is highly plastic and can be modulated both by pre- and post-dispersal environmental cues such as temperature, light, and to a lesser extent, nitrate [8–13]. In particular, low temperatures during seed production dramatically increase dormancy in *A. thaliana* [14–17] as well as in other species such as wheat [18] and wild oat [19].

Central to this temperature-dependent process in *A. thaliana* is the upregulation of a major genetic determinant of seed dormancy variation, *DELAY OF GERMINATION1* (*DOG1*) [14, 15, 20]. The *DOG1* locus exhibits genetic signatures suggestive of local adaptation, and field experiments have emphasized its pivotal role in controlling the timing of germination in wild *A. thaliana* populations [21–24]. Independently of their action on *DOG1*, low seed maturation temperatures induce deep dormancy by increasing the abscisic acid (ABA) / gibberellins (GA) ratio, two antagonistic phytohormones repressing and activating germination, respectively [14, 15]. Finally, low temperatures can promote coat-imposed dormancy by altering seed coat permeability through the upregulation of the flavonoids biosynthesis pathway, both during seed production [25] and vegetative phase [26].

Thus, it is clear that the induction of primary dormancy is regulated by both genetic and environmental factors, and the fact that distinct genotypes differ in their response to low temperatures indicates that genotype-environment interactions partly control the trait [17, 17, 27–29]. A direct consequence of this plasticity is that similar germination trajectories, defined as the evolution of the germination phenotype over time, can be promoted by different combinations of genotypes and environments. For example, strong *DOG1* alleles combined with a warm maternal environment and weak *DOG1* alleles combined with a cold maternal environment can both lead to highly dormant phenotypes [27].

Field studies have demonstrated that the maternal environment contributes greatly to seed dormancy variation under natural conditions [30]. Thus, given the existence of strong selection for timing of germination [22–24, 31], it has been speculated that the temperature-dependent regulation of primary dormancy is adaptive. For example, it may provide the mother plant with information regarding the seasonal environment, information that can be used to set progeny dormancy appropriately [32]. In addition, this mechanism is expected to enable bet-hedging strategies, in which seeds from the same population—or plant—express various dormancy phenotypes and germinate throughout the year to maximize fitness in unpredictable environments [33–36].

Although genotype-environment interactions have previously been reported to influence dormancy and germination plasticity in *A. thaliana* [27–29], the extent of this phenomenon and whether it is universal at the species level is unknown. Moreover, the genetic basis of the differential response to seed maturation temperatures, and more generally, of G x E variation, remains to be thoroughly investigated. Here, by growing a set of *A. thaliana* lines from Sweden in two different environments, we assess the effect of maternal temperature on seed dormancy in a local sample, before performing a GWAS to identify the genes responsible for the observed variation.

## Materials and methods

### Plant material and phenotyping

The 92 Swedish lines used in this study (S1 Table) were kindly donated by Joy Bergelson (University of Chicago) and were previously described in [22, 37]. Upon reception, lines were bulk propagated for one generation under standard lab conditions to minimize potential maternal effects. To produce seeds used in germination assays, six biological replicates of each genotype were first vernalized for eight weeks (4°C, 16 h day / 8 h night, 90% humidity). Then, three randomly chosen replicates were placed in a warm environment (21°C day / 16°C night), while the other three received a cold treatment (15°C day / 10°C night). Both treatments were applied from rosette stage to ripening and seed harvest. Seeds were harvested when about 50% of the siliques of a given plant had come to maturity and were subsequently placed in dry environment for after-ripening (30% relative humidity, 16°C, dark). The germination rate of seeds (percentage of germinated seeds) after-ripened for 21, 63 and 105 days (GR21, GR63 and GR105) was estimated for each genotype following standard methods [22, 38]. Briefly, about 75 seeds per genotype were spread on wet filter paper (Whatman, ref. 1001-047) in Petri dishes (Greiner Bio-One, ref. 628102) that were subsequently placed in moisture chambers to maintain high, constant humidity (close to 99%). Moisture chambers consisted of reasonably air-tight, transparent plastic boxes (Ikea), whose bottom was covered by ten layers of paper towel imbibed with tap water. Germination was scored by observing radicle emergence after a week of incubation at 23°C in standard long days (16 h day / 8 h night) and under fluorescent light (Osram L 58W 840 Lumilux, 40 *μ*m/m^2^/sec).

### Broad sense heritability

Broad sense heritability (H) was calculated for each dormancy phenotype as the genotypic variance divided by the total variance. Both variances were estimated using a linear mixed-model from the *lme4* package in R environment [39]. The model was as follows:

$$\begin{array}{c} Y=GEN+REP+e \end{array}$$ (1)

where *Y* is phenotype (germination rate), *GEN* is genotype (line), *REP* is technical replicate, and *e* is error. All variables were fitted as random effects.

### Variance component analysis

The variance component analysis was described earlier in [40]. It was carried out in LIMIX [41] using the following model:

$$\begin{array}{c} Y=\left[\mu_{warm},\mu_{cold}\right]⊗1_{N,1}+U_{global}+\psi \end{array}$$ (2)

where *μ*_*warm*_ and *μ*_*cold*_ are environment specific mean values, *U*_*global*_ is a matrix of global relatedness fitted as random effect, and *ψ* is noise.

### Genome-wide association mapping

Genome-wide scans were performed on arcsine transformed mean phenotypic values (germination rate) using a mixed-model accounting for population structure and 3,333,502 SNPs (≈ 266 SNPs / kb) derived from the 1001 genomes project [42–44]. The analysis was carried out on 88 lines using the GWA-Portal (https://gwas.gmi.oeaw.ac.at/; [45]), and both the settings and the results are fully browsable online (https://goo.gl/dt53nc). In this manuscript, rare SNPs [minor allele frequency (MAF) lower than 14%] were filtered out to minimize the risk of spurious associations (clearly amplified by the small population size) and to identify common variants that are more likely to explain the global pattern of variation observed across Sweden. We note that interactive plots displaying *P* values for rare SNPs (MAF lower than 14%) are available online (https://goo.gl/dt53nc). A 5% genome-wide significance threshold was determined using Bonferroni correction. However, because this correction is conservative when used in the context of GWAS, all marginally significant markers with a *P* value lower than 10^-6^ were considered of interest and grouped into peaks. A given peak had a ‘specific’ effect when its highest score [-log_10_(*P* value)] for any of the three warm phenotypes did not exceed 2, or a ‘common’ effect when its score for at least one phenotype in both environment was higher than 4. Peaks that did not meet any of these arbitrary criteria were regarded as having an ‘unclear’ effect. Finally, we considered all genes located between peaks borders plus-minus 20 kb when looking for candidate genes.

### Gene enrichment analyses

The seed dormancy *a priori* candidate gene list (91 genes; S2 Table) was built regardless of the GWAS results by searching the literature (mainly [15, 46]) and by querying the ARAPORT11 database using the following GO terms: GO:0048838 ‘release of seed from dormancy’, GO:1902039 ‘negative regulation of seed dormancy process’, and GO:0010162 ‘seed dormancy process’. This list is non exhaustive and we only included major dormancy regulators as well as seed specific genes affected by temperature during seed maturation. A gene was considered significantly associated when at least one SNP located 20 kb upstream or 20 kb downstream its coding sequence had a *P* value lower than 10^-4^. Lists of non *a priori* significantly associated TAIR11 genes were built in a similar manner for each phenotype, and the overrepresentation of *a priori* genes in the resulting lists was assessed using one-sided Fisher’s exact test as described in [47]. More specifically, we used a 2 x 2 contingency table in which row entries consisted of significant and non significant genes while column entries consisted of *a priori* and non *a priori* genes, which gave for categories: significant *a priori* genes, non significant *a priori* genes, significant non *a priori* genes and non significant non *a priori* genes. This analysis was performed in R environment [39] using the built-in ‘fisher.test()’ function, with the alternative parameter set to ‘greater’ as we tested for overrepresentation.

## Results

### Seed dormancy variation in the Swedish population

**Fig 1:**
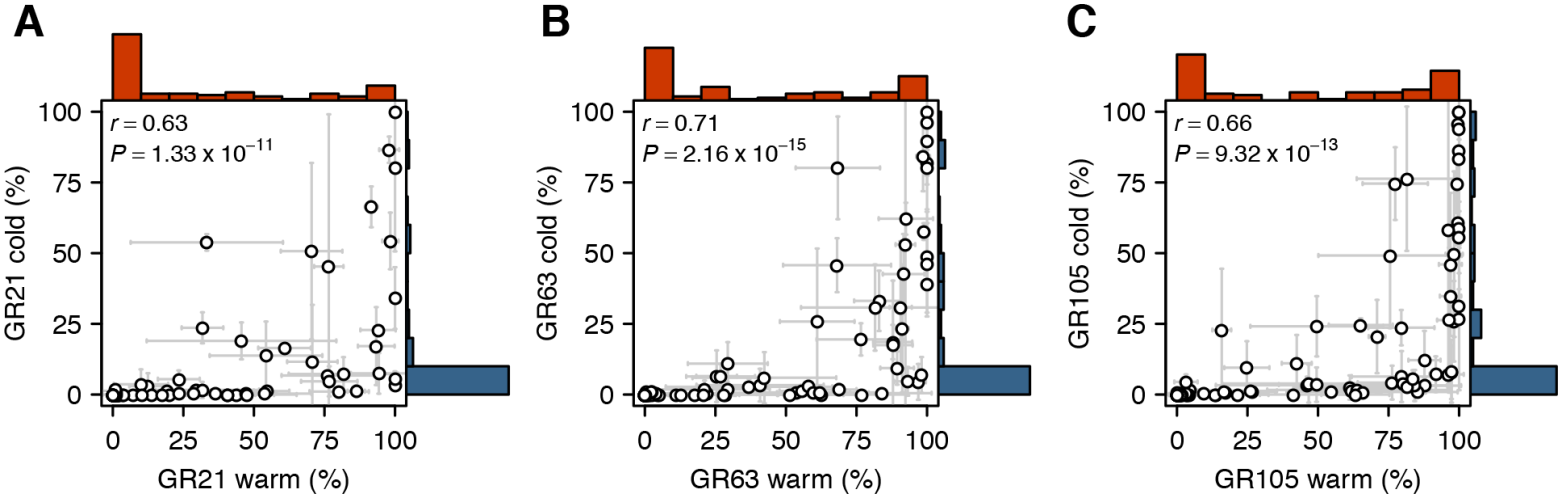
The effect of seed maturation temperature on seed dormancy variation. Scatter plots and histograms showing the relationship between dormancy traits as well as their phenotypic distribution. Seeds were produced either under warm (21°C; red) or cold (15°C; blue) conditions and after-ripened for either (A) 21, (B) 63 or (C) 105 days. Error bars represent the standard deviation within genotypes (n = 3, in few cases n = 2).

As expected, genotypes that experienced warm maternal temperature displayed great variation for seed dormancy, although more than one third of the lines remained dormant even after 105 days (Fig 1). In contrast, the vast majority of seeds produced at cold maternal temperature remained dormant throughout the experiment, in line with previous studies that have shown that a decrease in seed maturation temperature generally induces a deeper dormancy [14–17, 27, 28].

All six phenotypes were correlated, especially within temperature treatments (S1 Fig), and broad sense heritabilities were remarkably high (ranging from 0.85 to 0.94; Table 1), although they became moderate when considering lines with intermediate phenotypes only (GR ≥ 5% and ≤ 95%; ranging from 0.67 to 0.75; Table 1). To assess the relative effects of genes and the environment on dormancy variation, we performed a variance components analysis, in which we modelled the effect of genotype (G; line), environment (E; maturation temperature) and the interaction of both (G x E; line x maturation temperature) [40]. G and G x E effects contributed equally after 21 days (37% and 38%, respectively), but the purely genetic effect increased over time. Environment effects were responsible for 20% of the variance regardless of time point (Table 2). Although the accuracy of this analysis is limited because of the relatively small sample size (due to many of the tested lines being too dormant to be ‘informative’), these findings agree with previous studies and indicate that the dormancy variation observed in the Swedish sample is, to a large extent, explained by G x E effects [17, 27–29].

**Table 1:** Heritability of seed dormancy traits.

	H_warm_	n_warm_	H_cold_	n_cold_
**Full sample**				
**GR21**	0.93	92	0.85	92
**GR63**	0.93	92	0.87	92
**GR105**	0.94	92	0.87	92
**Intermediate lines**				
**GR21**	0.75	38	0.65	20
**GR63**	0.75	42	0.65	27
**GR105**	0.71	38	0.67	32

Broad sense heritabilities (H) were calculated using either the full sample or subsets of lines (whose number is indicated by ‘n’) with intermediate phenotypes (GR ≥ 5% and ≤ 95%).

**Table 2:** Genetic and environmental effects on seed dormancy variation.

	G	E	G x E	noise
**GR21**	37.27	20.48	38.13	4.13
**GR63**	50.06	19.46	19.18	11.30
**GR105**	47.75	22.33	24.86	5.06

### Genetic variation in the response to low seed maturation temperatures

It is thus clear that the effect of the maternal environment differs between Swedish genotypes. About one third of the mild- and non-dormant lines appeared to be relatively insensitive to the maternal environment after 21 days, and one line, Gro-3, reached 100% of germination in both conditions. In sharp contrast, other non-dormant lines such as Löv-1 and T480 were heavily affected by the maternal environment and displayed very low germination rates when seeds were produced at low temperatures, even after 105 days of after-ripening (S2 Fig).

**Fig 2:**
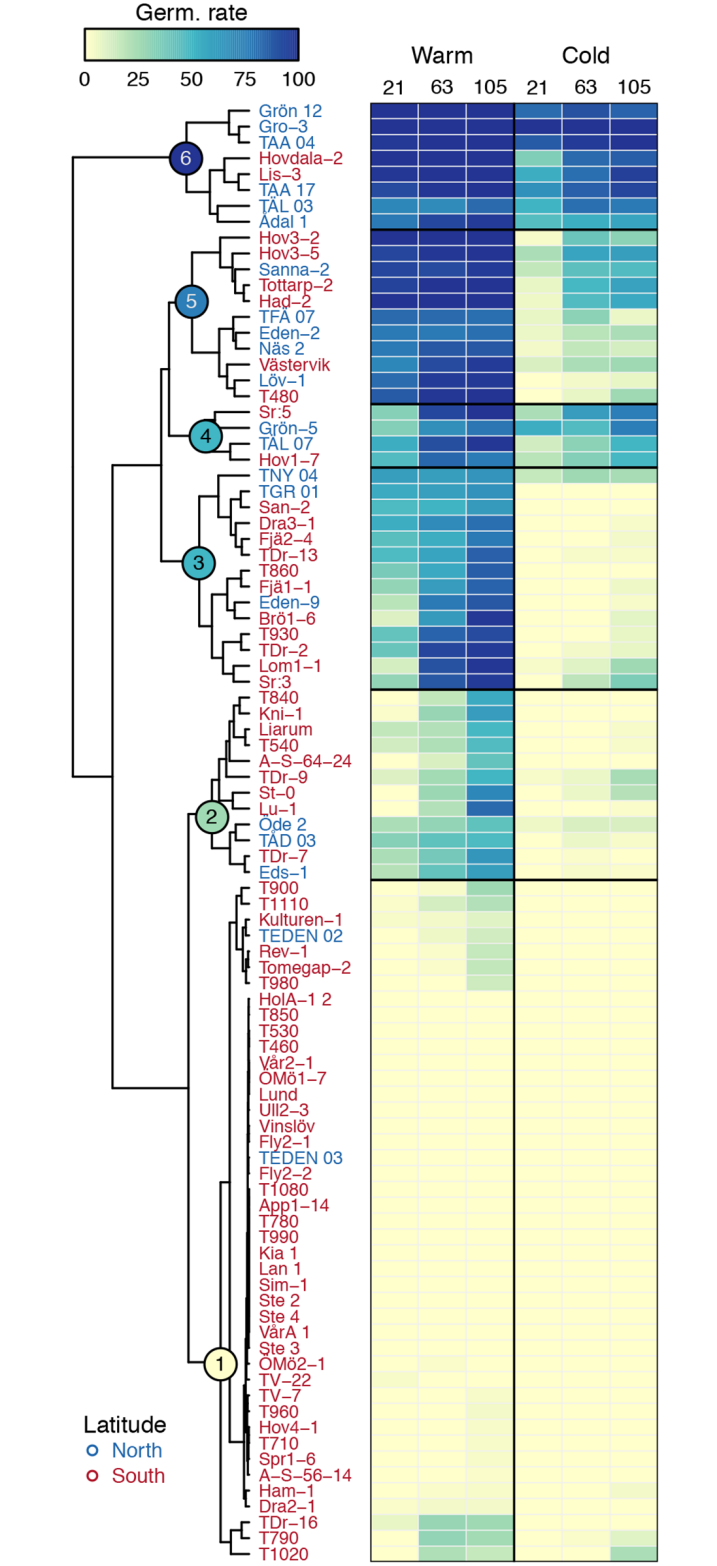
The effect of seed maturation temperature on germination trajectories. Clustering dendrogram reporting the high disparity in germination trajectories across maternal environments (warm or cold) and time (21, 63 or 105 days of after-ripening). The six major clusters are numbered from 1 to 6 and are indicated with colored circles on the nodes of the dendrogram. Lines names are colored according to latitude of origin: south Sweden (red) is defined as the region below 60°N and north Sweden (blue) as the region above 60°N. Heatmap colors represent germination phenotypes, with darker shades indicating higher germination rates.

To characterize the genetic variation in the response to low seed maturation temperatures, we clustered lines based on their germination phenotypes across environments and time. We identified six main clusters representing distinct germination behaviors (Fig 2). The largest cluster (cluster 1; n = 43) is not only deeply dormant but also insensitive to after-ripening, making it ‘non-informative’ in the sense that it is not possible to assess its degree of responsiveness to temperature. Two smaller clusters (clusters 2; n = 12, and 3; n = 14) display shallow to mild dormancy at 21°C that can be lifted with after-ripening, but the cold treatment induces deep dormancy that can not be broken. Two clusters (5 and 6, n = 11 and n = 8, respectively) are both non-dormant at 21°C, but cold seed maturation temperatures dramatically increased dormancy levels of the former, but had very little effect on the latter. This last observation clearly confirms that there is natural genetic variation in the response to low temperatures and that genotype-environment interactions underlie dormancy variation in the Swedish population. A spectacular example of this differential response can be found in the opposite trajectories of Gro-3 (cluster 6) and Löv-1 (cluster 5) (Fig 2 and S2 Fig). Finally, a small cluster (cluster 4; n = 4) shows low dormancy regardless of the maternal environment, demonstrating that the degree of responsiveness is independent of the dormancy level. [27] have previously shown that similar germination phenotypes and trajectories can be reached via different paths, and although we only assess the effect of pre-dispersal temperatures, our results go in the same direction. For instance, cluster 5 and to some extent cluster 3 are non-dormant when seeds are produced at 21°C, but lower seed maturation temperatures induced a deep dormancy, comparable to that of cluster 1.

### Geographic pattern of the response to low maturation temperatures

**Fig 3:**
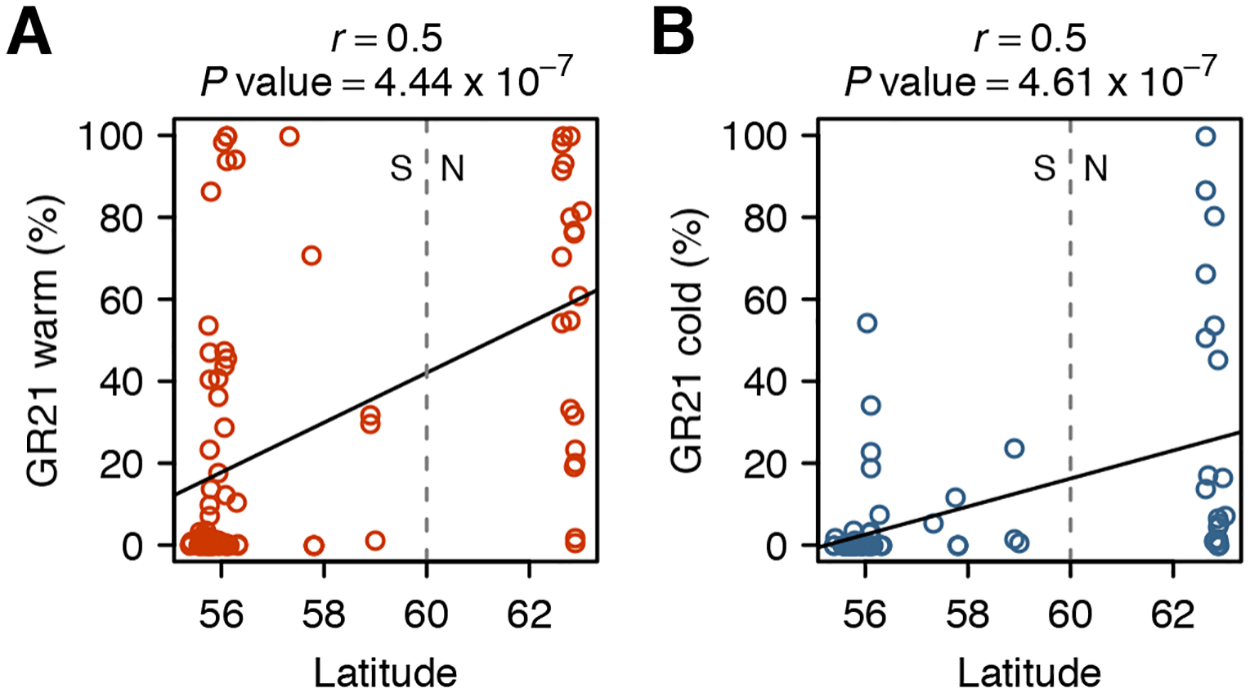
The geographic pattern of the dormancy variation. Correlation between latitude and either (A) GR21 warm or (B) GR21 cold. As in Fig 2, we define south Sweden (S) as the region below 60°N and north Sweden (N) as the region above 60°N. See S1 Fig for the correlations between latitude and the other dormancy phenotypes.

It is well established that seed dormancy in *A. thaliana* correlates with latitude and climate variables such as temperature and precipitation, with northern lines generally being less dormant than southern ones [21, 22, 48]. This geographic pattern, thought to reflect local adaptation, was also observed in this study: both GR21 warm (Fig 3A; *r* = 0.5, *P* = 4.44 x 10^-7^) and cold (Fig 3B; *r* = 0.5, *P* = 4.61 x 10^-7^) are correlated with latitude. However, we note that these relationships are not strict, possibly reflecting adaptation to microenvironmental variation.

When grown under cold maternal conditions, almost all non-dormant lines from the south appear to be severely affected, and exhibit strongly reduced germination rates. Northern lines, however, display a greater variation in their response to low seed maturation temperature, with some genotypes being insensitive (Fig 3B). This suggests that the response not only varies along a latitudinal gradient but also at a very local scale. These findings are nicely captured by the above-mentioned clustering approach, in which most lines from northern Sweden are binned in the sensitive and insensitive clusters 5 and 6, respectively (Fig 2).

### GWAS for the response to low seed maturation temperatures

**Fig 4:**
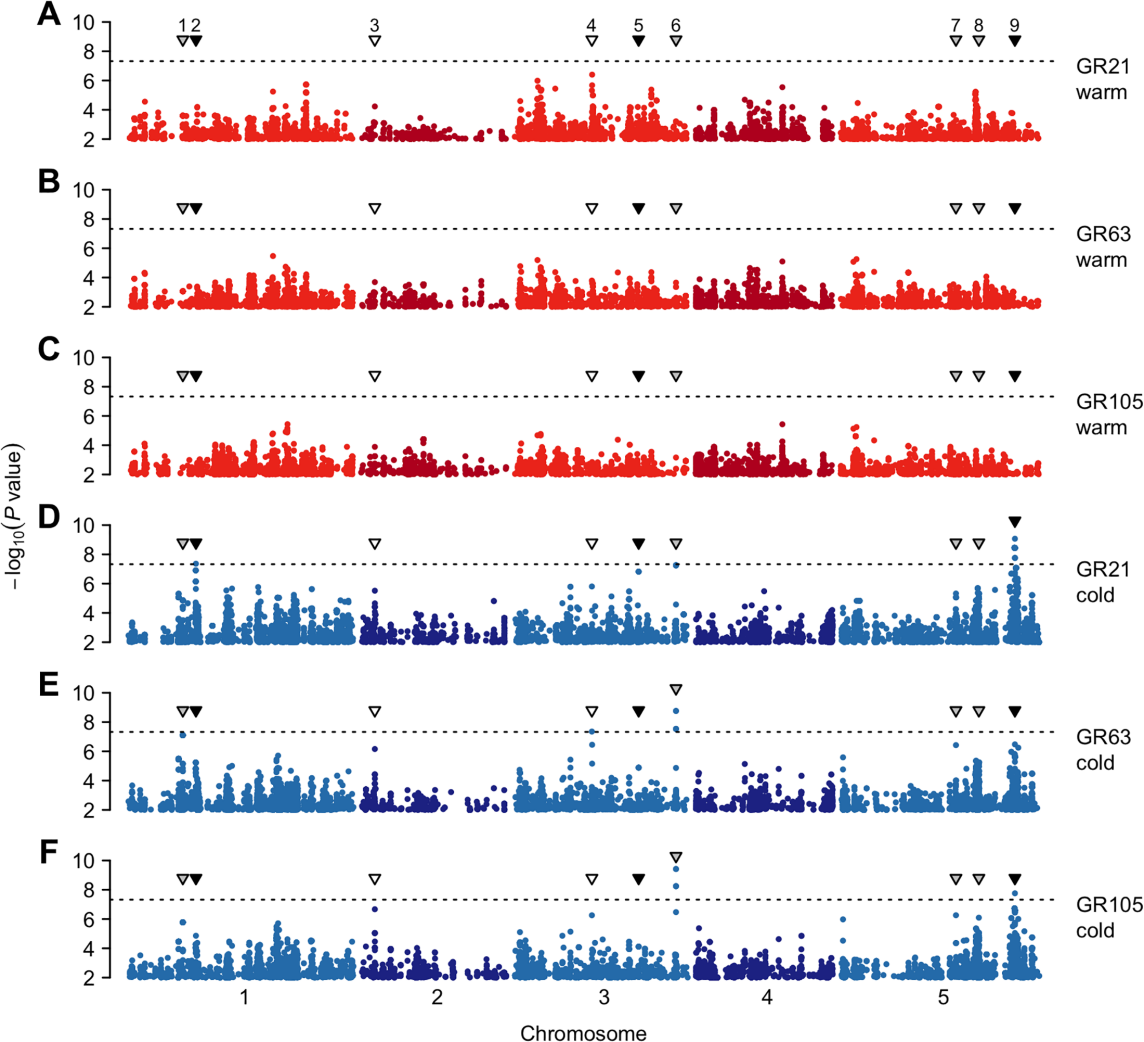
GWAS for seed dormancy traits. Manhattan plots of genome-wide association results for germination rate of seeds set either in (A-C) warm or (D-F) cold environments and after-ripened for (A and D) 21, (B and E) 63 or (C and F) 105 days. The dotted horizontal line indicates a significance level of 0.05 after Bonferroni correction for multiple testing. Triangles show the position of the nine peaks with *P* values < 10^-6^ for at least one phenotype. Triangle color indicates the type of effect: white, ‘common’; black, ‘specific’; grey, ‘unclear’. Are only displayed SNPs with a minor allele frequency ≥ 14%. The GWAS results are fully browsable online: https://goo.gl/dt53nc.

To uncover the polymorphisms underlying the differential response to low seed maturation temperatures, we assessed the significance of associations between the seed dormancy phenotypes and genome-wide SNP markers from the 1001 genomes project using a mixed-model accounting for population structure [42–44]. Four lines with missing genotype information were removed from the dataset, bringing the number of lines to 88 (S1 Table). GWAS results were very comparable between time points, as expected given the strong correlations between traits within treatments (S1 Fig), but they differed markedly between treatments, with no strong association for the warm phenotypes while several peaks reached genome-wide significance for the cold phenotypes (Fig 4).

To explore the GWAS results, we first performed an *a priori* gene enrichment analysis using a set of 91 genes with known or predicted function in seed dormancy regulation (S2 Table). No enrichment was detected for any of the six traits, but the fact that *DOG1* is the most strongly associated *a priori* candidate suggests that some of the associations are true signals rather than noise (S3 Table).

Next, we looked at the associations in greater detail, limiting ourselves to an arbitrarily chosen *P* value cutoff of 10^-6^. This yielded a total of nine regions across the six phenotypes, regions that we classified into three categories (see Material and methods): those with a ‘common’ effect (they tend to have a similar effect on both warm and cold phenotypes), those with a ‘specific’ effect (they tend to influence only the cold phenotypes), and last, those with an ‘unclear’ effect (Fig 4 and Table 3).

**Table 3:** GWAS for seed dormancy traits summary.

Peak	Chr.	Pos.	MAF	Warm			Cold			Effect	Number of genes	Candidate genes
				GR21	GR63	GR105	GR21	GR63	GR105			
1	1	7381921	0.28	3.60	3.06	2.47	4.86	7.10	5.77	unclear	10	*URGT2*
2	1	9148998	0.14	0.33	0.47	0.46	**7.36**	5.21	4.86	specific	25	*SNS1*
3	2	1882558	0.26	4.23	3.69	3.89	5.52	6.16	6.67	common	12	*–*
4	3	10504938	0.22	5.32	3.79	2.57	5.80	**7.35**	6.26	common	17	*–*
5	3	16820806	0.14	1.97	1.83	1.39	6.82	4.89	4.11	specific	8	*PHOT1*
6	3	21867111	0.15	3.19	3.36	2.39	7.24	**8.76**	**9.42**	unclear	10	*–*
7	5	15630623	0.14	2.80	3.27	2.88	5.32	6.42	6.26	unclear	10	*–*
8	5	18726653	0.20	2.96	2.06	1.58	4.99	5.21	6.09	unclear	11	*–*
9	5	23596831	0.14	1.22	1.80	1.75	**9.07**	6.48	**7.76**	specific	34	*SIP1, PHOT2*

Are listed top SNPs for the nine GWAS peaks with a score [-log_10_(*P* value)] ≥ 6 for at least one phenotype. Genome-wide significant scores (≥ 7.33; Bonferroni correction) are highlighted in bold. The number of genes encompassed by each peak is also indicated.

The only two associations with a ‘common’ effect, peaks 3 and 4, lie on chromosomes 1 and 2, respectively, but no clear candidate could be identified among the genes tagged by those peaks (S4 Table). The major ‘specific’ hit on chr. 5 (peak 9) falls directly in *SOS3-INTERACTING PROTEIN 1* (*SIP1*), a gene encoding a SnRK3-type protein kinase likely to be involved in stress and ABA signalling [49, 50]. However, as the peak is quite broad (more than 100 kb; see S3 Fig), we also examined the 33 other genes present in the associated region (S4 Table). Among those was *PHOTOTROPIN2* (*PHOT2*), a promising candidate not only because of its role in the photoregulation of germination [51], but also because *PHOT1*, a gene with similar function, was identified on chr. 3 below peak 5 (also ‘specific’). The third ‘specific’ association, peak 1, colocalizes with *SnRK2-substrate 1* (*SNS1*), a gene required in ABA signalling [52]. Among the four remaining peaks with an ‘unclear’ effect, we note the presence of *URGT2*, a seed specific gene controlling mucilage formation (chr. 1, peak 2) [53]. Finally, in contrast with our previous work [22], no strong association was detected at the *DOG1* locus (peak 8, see S4 Fig), a point we discuss below.

## Discussion

The regulation of seed dormancy by maternal environment temperature has been described in numerous plant species and appears to be conserved among higher plants [12]. In *A. thaliana*, the underlying mechanism has mainly been studied at the molecular level, using very specific, often artificial backgrounds [17, 27]. Few studies have approached this temperature-dependent regulation from a natural variation perspective, and both its extent and genetic basis remain unknown. Here, by focusing on a set of Swedish lines, we aimed to characterize this phenomenon at the population level and to identify its underlying genetic basis.

### The role of maternally-regulated dormancy in plant adaptation

Despite the prevailing deep dormancy in the Swedish sample, several lines let us assess the effect of maternal temperature on seed dormancy variation (Fig 1). In agreement with previous reports [17, 27, 28], we find that, although low seed production temperatures generally increase primary dormancy, the effect differs between lines, indicating that the trait is influenced by genotype-environment interactions. This observation is further supported by a variance component analysis, which estimates that almost 40% of the dormancy variation in the Swedish sample is due to G x E effects (Table 2; GR21). This, and the fact that high G x E variation was previously observed in a set of world-wide lines [28], suggests that the maternal regulation of seed dormancy by environmental cues is conserved not only at the population, but also at the species level.

By combining different genotypes and seed maturation temperatures, [27] have demonstrated that identical germination trajectories can be achieved by going down different paths. Likewise, we found that cold seed maturation temperatures can produce highly dormant phenotypes, similar in depth to those caused by genetic effects, showing that environmental variation can have large repercussions on the expression of genetic variation (Fig 2).

Because the timing of germination is one of the major fitness components in *A. thaliana* [22, 23], the idea that such maternal regulation may be adaptive is attractive, although the rationale for its existence in this species is yet to be established [27]. In Sweden, where *A. thaliana* mainly behaves as a winter annual, seed dispersal usually occurs in spring, and germination in fall. Therefore, we hypothesize that Swedish populations use ambient temperatures to fine-tune the depth of primary dormancy, should flowering happen earlier or later in the season. This is especially true in northern Sweden, where plants vernalize before winter [54] and usually flower as soon as the snow melts (daylength and temperature permitting), the timing of which is likely to vary from year to year.

On the other hand, it is difficult to make sense of the great variability in the response to low temperatures observed among northern lines (Figs 2 and 3B), as one would expect low dormancy levels to be necessary to make the most of an extremely short growing season (which is the norm at these latitudes). This suggest that these lines, despite their common geographical origin and high vernalization requirement [54, 55], have different germination phenologies. Alternatively, although modelling approaches predict that reproduction occurs under similar temperatures across the species range [56], it is possible that populations from northern Sweden set seeds in slightly warmer temperatures [57], which would diminish the environmental effect and result in weaker dormancy.

Germination in Sweden also happens—to a much lesser extent—in spring and/or summer, as it is not uncommon to observe flowering plants at different times of the year in some southern Swedish populations (Kerdaffrec and Nordborg, personal observations). This could be evidence of bet-hedging, and it is clear that, in this case, the ability to adjust dormancy levels through maternal regulation according to seasonal environment would be advantageous. However, a constant monitoring of these populations across several years would be necessary to rule out the possibility that distinct genotypes expressing different life cycles segregate at these locations.

### The genetic basis of the response to low seed maturation temperatures

Although the molecular mechanisms involved in the temperature-dependent maternal regulation of seed dormancy are being revealed, its underlying genetic basis has not been studied yet. Here, by performing a GWAS on six dormancy traits, we identify a total of nine distinct associations, three of which have a ‘specific’ (i.e., interaction) effect (Table 3). SNPs within these three peaks are associated with high germination rates in response to cold seed maturation temperatures, which suggests that they tag genes involved in the temperature-dependent regulation of dormancy.

Among the candidates for the ‘specific’ genes, we identified *PHOT1* and *PHOT2*, which both encode phototropins that mediate several light-dependent processes such as hypocotyl phototropism [58], stomatal opening [59] and germination [51]. Light, along with temperature, is one of the factors regulating primary dormancy induction, and later in the soil seed bank dormancy release and germination. Light-induced germination is mainly promoted by phytochromes, especially *PHYB* and *PHYA* [60–62], and phototropins are assumed to act downstream of them, by modulating the germination response via the integration of light and temperature signals [51]. Interestingly, *TRANSPARENT TESTA 12* (*TT12*), a gene central to the induction of coat-imposed dormancy in response to low seed maturation temperatures [25], was identified earlier in a GWAS for germination traits under various light treatments [63]. This stresses the point that light and temperature signalling pathways may interact both during the induction and the release of dormancy. Therefore, it is possible that *PHOT1* and *PHOT2* play a role in dormancy regulation, direct evidence of which remains to be established. Finally, it should be mentioned that *TT12* and *PHYTOCHROME-INTERACTING FACTOR-LIKE 6* (*PIL6*), a gene negatively regulating *PHYB* [64], are located 45 kb and 35 kb downstream of the strongest association detected in our analysis (peak 6, chr.3), respectively.

Although our GWAS identified compelling candidates, we emphasize that most signals are driven by the same few lines, a consequence of the small sample size and the limited phenotypic variation. Indeed, most of the associated SNPs, and especially those with a ‘specific’ effect, are often private to a small subset of non-dormant, cold-temperature-insensitive northern lines (Table 3). Some of these associations may also be false positives due to confounding by population structure, although quantile-quantile plots do not show extremely inflated *P* values (S5 Fig).

As previously mentioned, both the power and the resolution of our GWAS are undermined by the limited dormancy variation observed among Swedish lines. Conspicuously, almost half of the tested lines are deeply dormant (‘non-informative’ lines). In future experiments, it could be interesting to focus only on lines with mild- or non-dormant phenotypes, or alternatively, to apply variable cold stratification treatments to gradually alleviate dormancy and maximize the variation. On the other hand, classical quantitative trait locus (QTL) mapping could be performed in segregating populations derived from contrasted lines such as, for example, Gro-3 (insensitive) and Löv-1 (sensitive) (S2 Fig).

Finally, we have previously shown by performing a GWAS on 161 Swedish lines that *DOG1* is the major regulator of seed dormancy in Sweden [22]. The *DOG1* region was also associated in the present study (S4 Fig), but to a lesser degree, although similar phenotypes were used in both cases (GR21 warm). There are two likely reasons for this discrepancy. First, the phenotypes are not perfectly correlated (Pearson’s *r* = 0.84) and several lines were slightly more dormant in this study than in the previous (S6 Fig), reflecting the plastic nature of seed dormancy. Secondly, even if the previously identified *DOG1* alleles segregate among the lines used here, a different sample size is likely to give different results because of the pitfalls intrinsic to GWAS (altered power, changes in allele frequencies, epistasis, among others) [65]. As a demonstration, a GWAS on both GR21 warm phenotypes (previous and present) using the exact same set of lines (86, the overlap between both studies) gave very similar results at the *DOG1* locus (S7 Fig).

## Conclusion

In this study, we confirm that the maternal environment interacts with genotype in controlling seed dormancy variation and characterize this interaction in a natural variation context, at the population level. Our GWAS results, in spite of their limitations, agree with the fact that the maternal environment impacts the genetic basis of seed dormancy, although functional evidence is required to validate these findings and confirm the role of the identified candidates.

Because the genes and pathways involved in the regulation of environmentally-dependent transitions are starting to be well characterized, it will become increasingly possible to integrate them into predictive models that, ultimately, could be validated in the field. This should lead towards a better understanding of the molecular and genetic basis of genotype-environment interactions, which is not only important to evolutionary biology [66, 67] but also to modern agriculture, especially in the light of climate change [68–70].

## Supporting information

S1 Fig Pairwise correlations between seed dormancy traits and latitude. Lower panel: pairwise scatter plots showing the relationships between variables; Upper panel: pairwise Pearson’s correlation coefficients between variables. All correlations presented here are significant (*P* < 0.05).
(TIF)

S2 Fig The diverse germination trajectories in Sweden. Replicates are represented using different types of dashed lines while plain lines indicate mean values. Red and blue lines correspond to warm (21°C) and cold (15°C) maternal environments, respectively.
(TIF)

S3 Fig Enlarged view of the region surrounding peak 9 (1 Mb). Local manhattan plots for (A-C) GR21-GR105 warm and (D-F) GR21-GR105 cold. Triangles in (A) denote the *PHOT2* and *SIP1* locus and SNP color reflects the extent of linkage disequilibrium (LD) starting from the most strongly associated SNP in the region at position 23,596,831 on chromosome 5.
(TIF)

S4 Fig Enlarged view of the region surrounding peak 8 (1 Mb). Local manhattan plots for (A-C) GR21-GR105 warm and (D-F) GR21-GR105 cold. Triangle in (A) denotes the *DOG1* locus. SNP color reflects the extent of LD starting from the most strongly associated SNP in the vicinity of the *DOG1* locus at position 18,580,359 on chromosome 5.
(TIF)

S5 Fig Quantile-quantile plots of GWAS *P* values. Comparisons of the expected and observed -log_10_(*P* value) from GWAS for GR21-GR105 warm (A-C, red) and GR21-GR105 cold (D-F, blue). Are only displayed common SNPs (minor allele frequency ≥ 14%).
(TIF)

S6 Fig The relationship between present and previously published GR21 phenotypes. (TIF)

S7 Fig *DOG1* region association scans for GR21 warm phenotypes. Local scans for (A) the GR21 warm phenotype from the present study and (B) the previously published GR21 warm phenotype [22]. The exact same set of lines (86, the overlap between both studies) was used for the local scans.
(TIF)

S1 Table List of the 92 Swedish accessions used in this study. Accessions that were excluded from the GWAS are marked with ‘0’ in the ‘GWAS panel’ column.
(CSV)

S2 Table List of *a priori* seed dormancy genes used in this study. (CSV)

S3 Table Top associated *a priori* seed dormancy genes. Seed dormancy genes with a score [-log_10_(*P* value)] ≥ 4 for at least one phenotype.
(CSV)

S4 Table GWAS for seed dormancy traits full summary and associated genes. (CSV)
