# Supplementary figures and images for: The maternal environment interacts with genetic variation in regulating seed dormancy in Swedish Arabidopsis thaliana
Source: PLoS One. 2017 Dec 27;12(12):e0190242. doi: 10.1371/journal.pone.0190242 (PMC5744996; doi:10.1371/journal.pone.0190242)

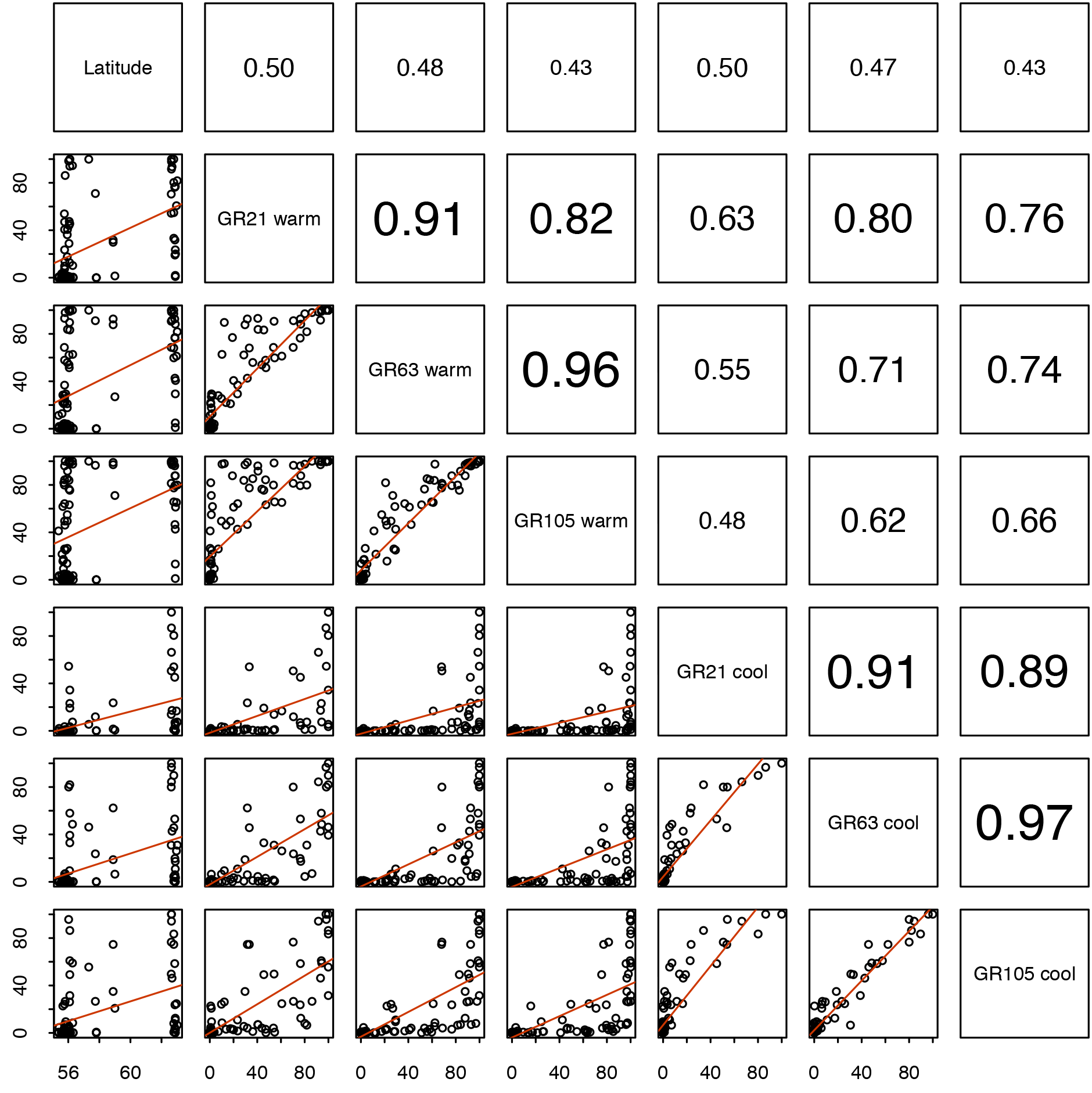

Supplement: S1 Fig — Lower panel: pairwise scatter plots showing the relationships between variables; Upper panel: pairwise Pearson’s correlation coefficients between variables. All correlations presented here are significant (P < 0.05). (TIF) [file pone.0190242.s001.tif]

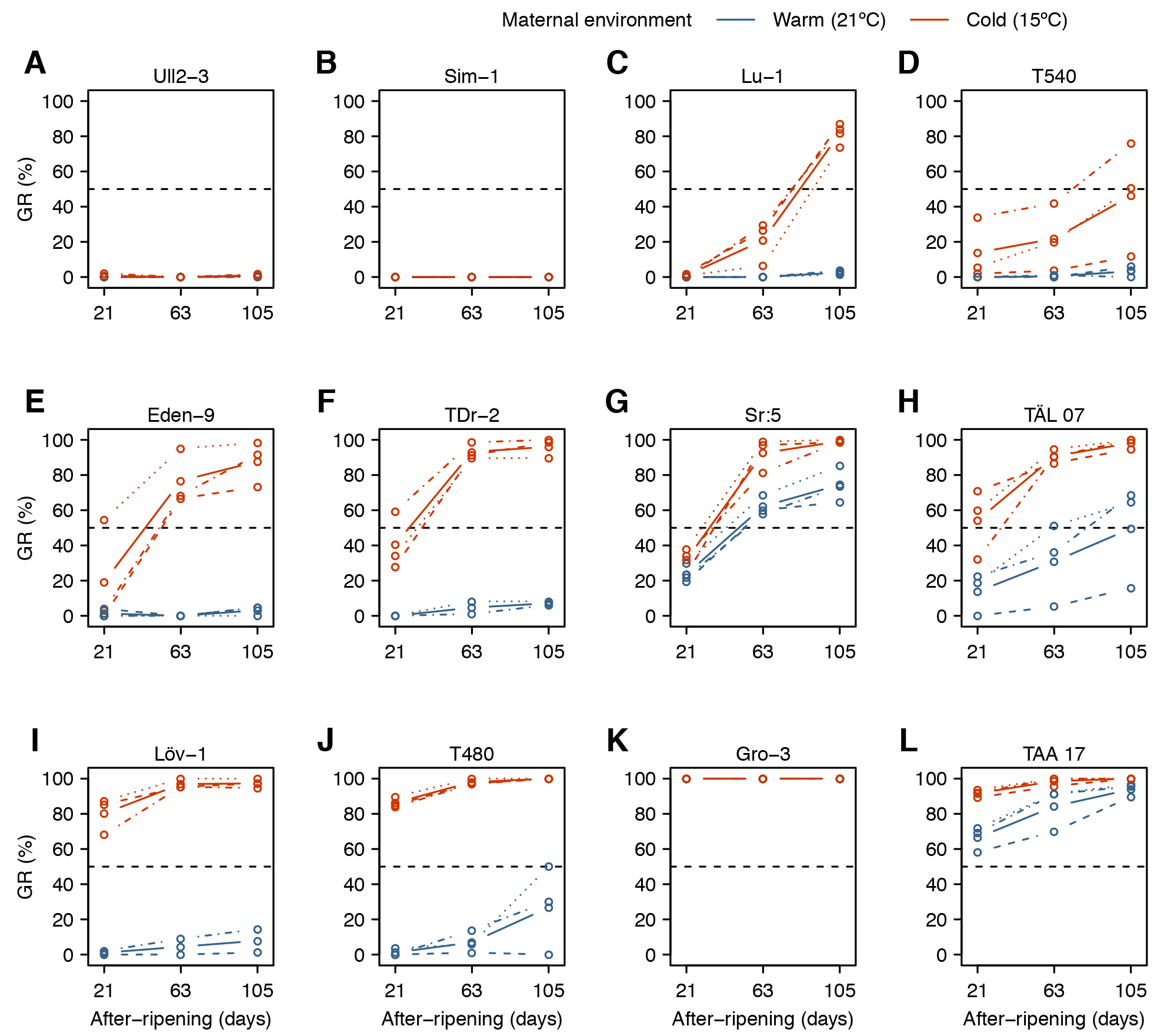

Supplement: S2 Fig — Replicates are represented using different types of dashed lines while plain lines indicate mean values. Red and blue lines correspond to warm (21°C) and cold (15°C) maternal environments, respectively. (TIF) [file pone.0190242.s002.tif]

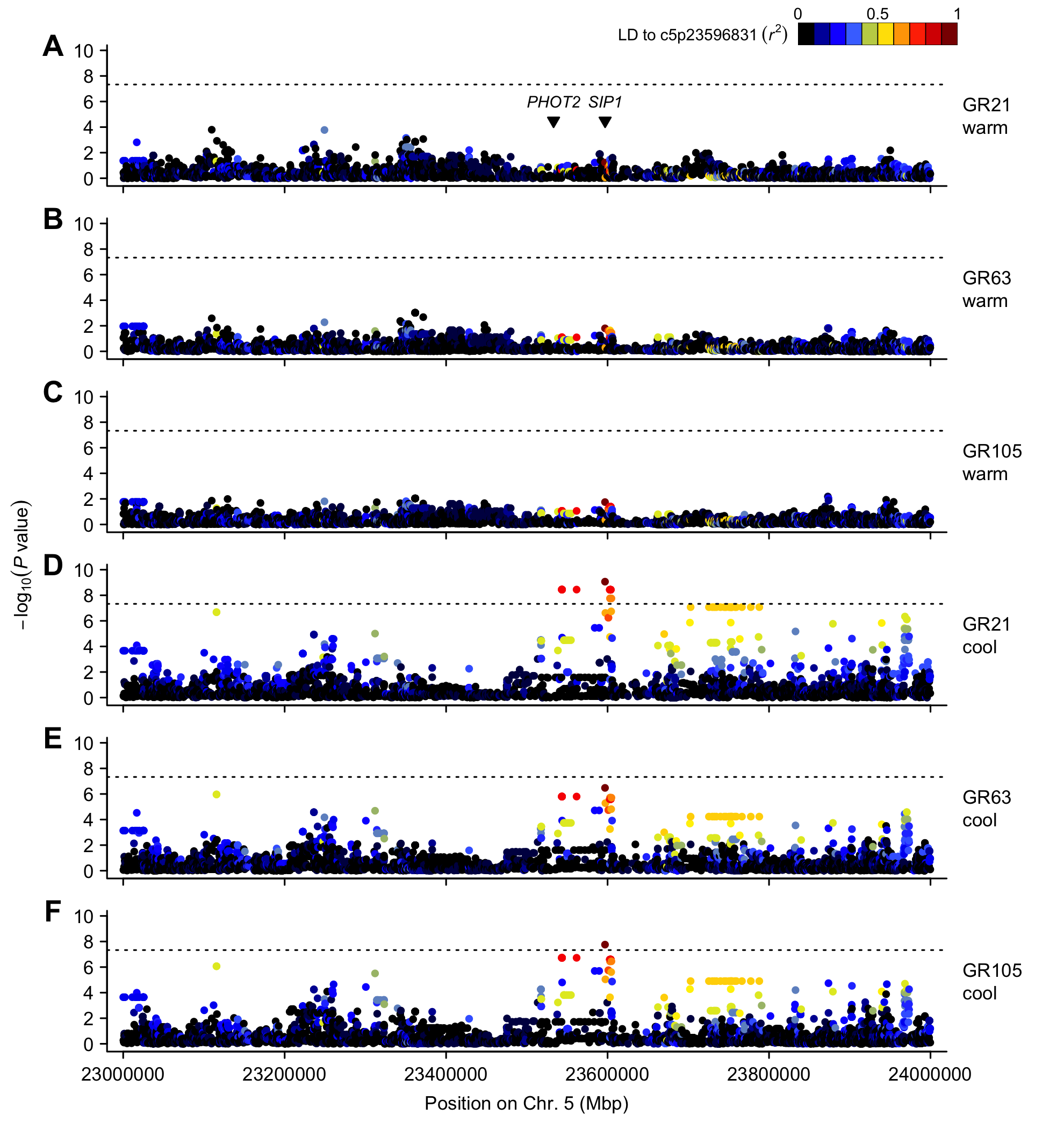

Supplement: S3 Fig — Local manhattan plots for (A-C) GR21-GR105 warm and (D-F) GR21-GR105 cold. Triangles in (A) denote the PHOT2 and SIP1 locus and SNP color reflects the extent of linkage disequilibrium (LD) starting from the most strongly associated SNP in the region at position 23,596,831 on chromosome 5. (TIF) [file pone.0190242.s003.tif]

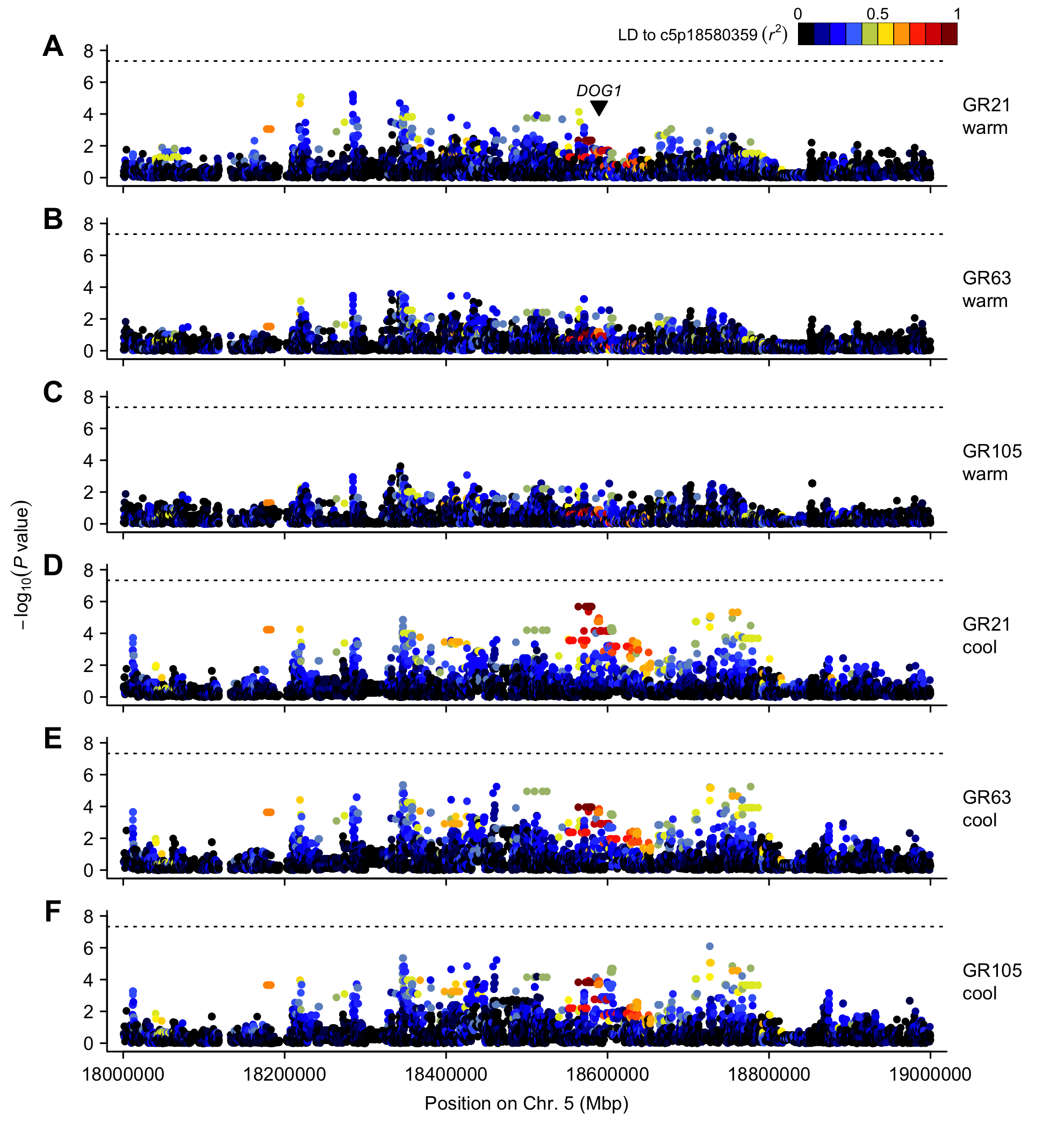

Supplement: S4 Fig — Local manhattan plots for (A-C) GR21-GR105 warm and (D-F) GR21-GR105 cold. Triangle in (A) denotes the DOG1 locus. SNP color reflects the extent of LD starting from the most strongly associated SNP in the vicinity of the DOG1 locus at position 18,580,359 on chromosome 5. (TIF) [file pone.0190242.s004.tif]

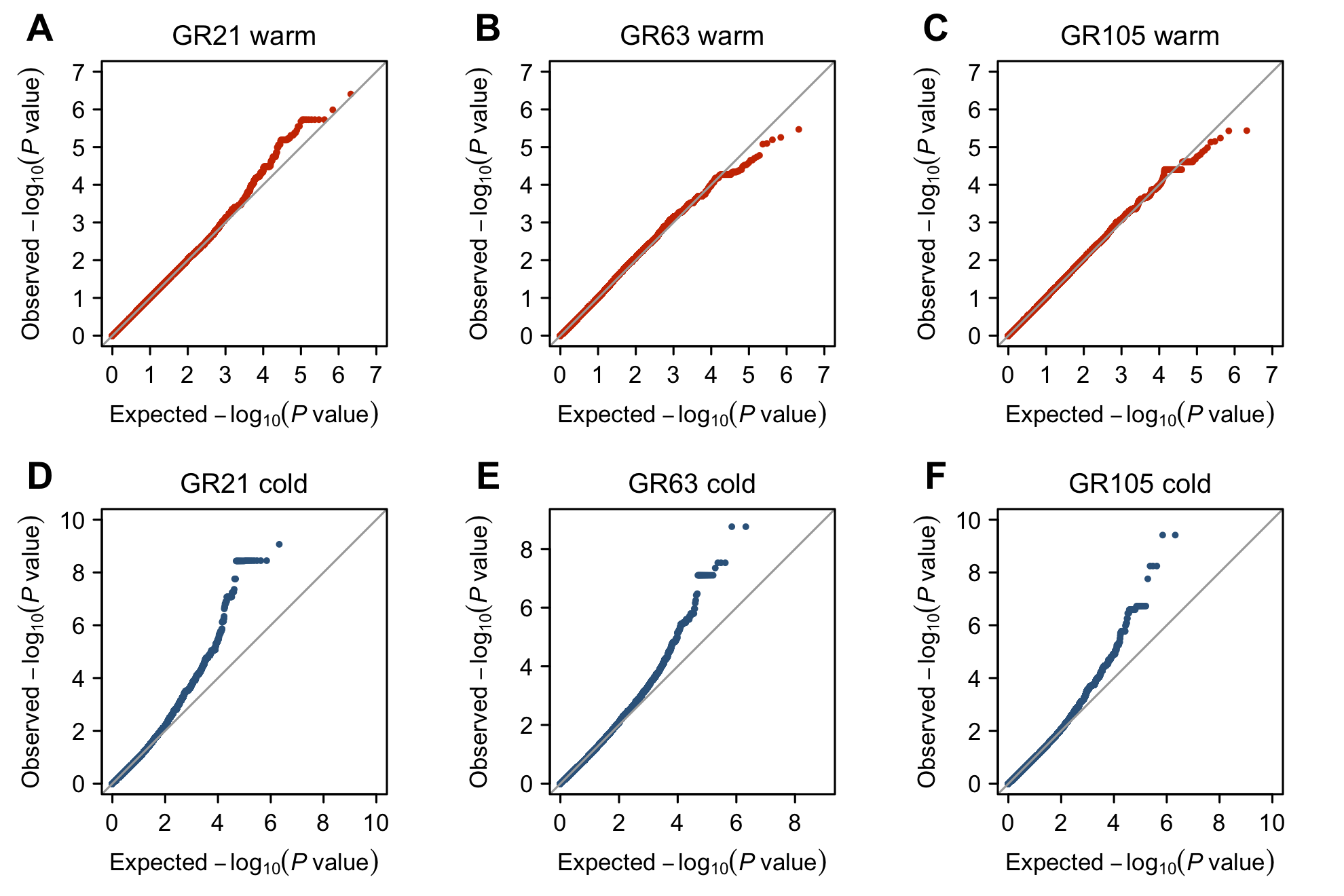

Supplement: S5 Fig — Comparisons of the expected and observed -log10(P value) from GWAS for GR21-GR105 warm (A-C, red) and GR21-GR105 cold (D-F, blue). Are only displayed common SNPs (minor allele frequency ≥ 14%). (TIF) [file pone.0190242.s005.tif]

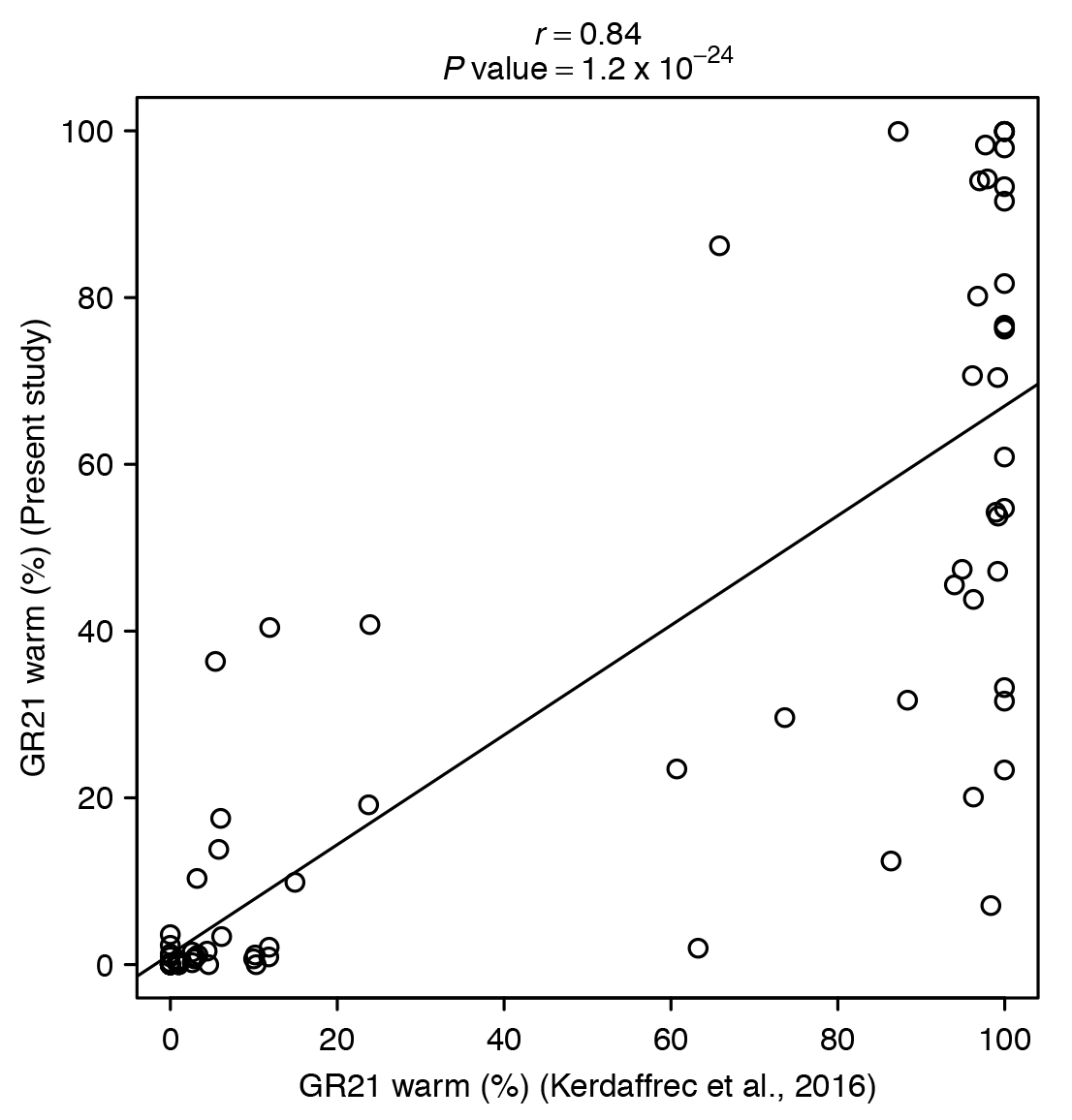

Supplement: S6 Fig — (TIF) [file pone.0190242.s006.tif]

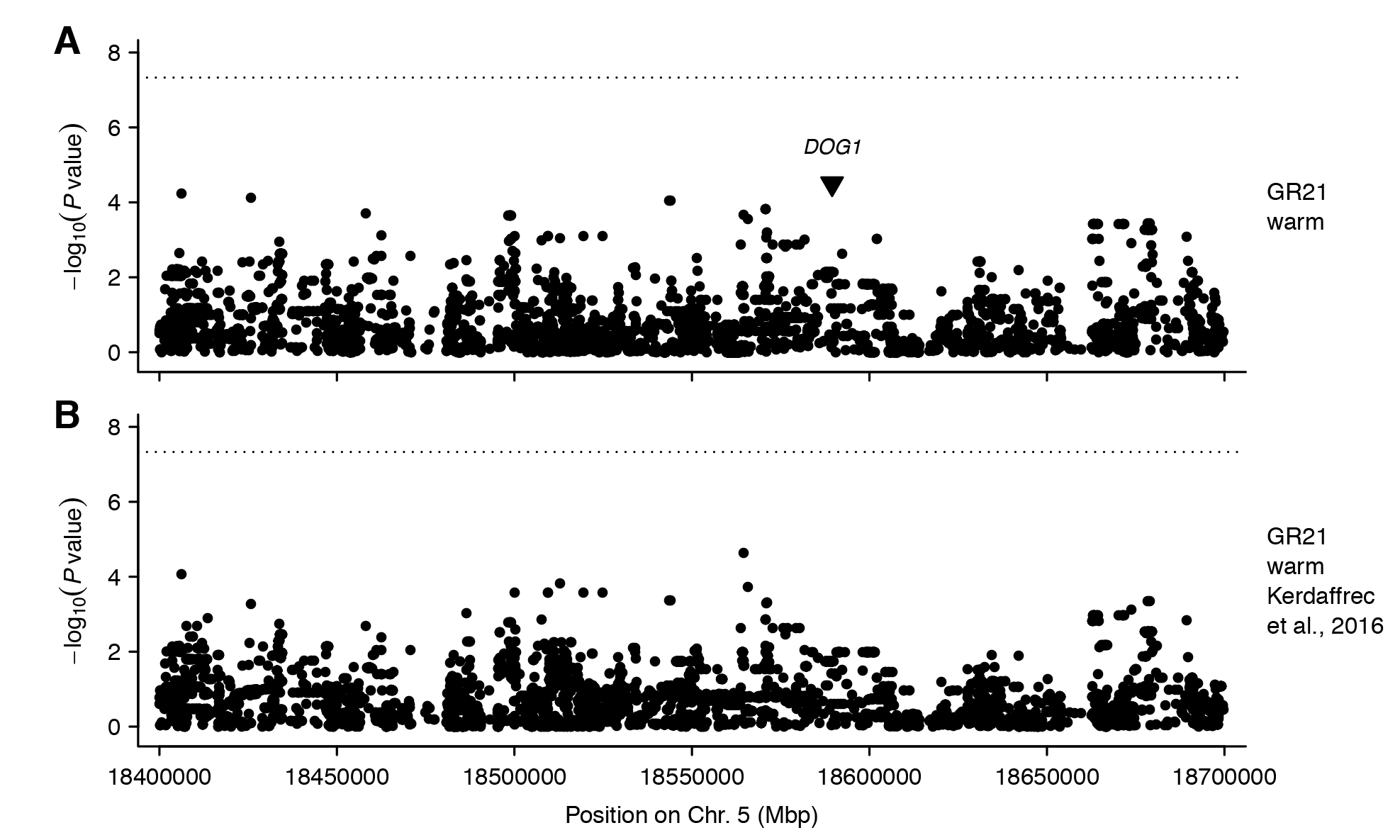

Supplement: S7 Fig — Local scans for (A) the GR21 warm phenotype from the present study and (B) the previously published GR21 warm phenotype [22]. The exact same set of lines (86, the overlap between both studies) was used for the local scans. (TIF) [file pone.0190242.s007.tif]
